# *Grifola frondosa* (Maitake) Extract Reduces Fat Accumulation and Improves Health Span in *C. elegans* through the *DAF-16/FOXO* and *SKN-1/NRF2* Signalling Pathways

**DOI:** 10.3390/nu13113968

**Published:** 2021-11-07

**Authors:** Paula Aranaz, Adriana Peña, Ariane Vettorazzi, María José Fabra, Antonio Martínez-Abad, Amparo López-Rubio, Joan Pera, Javier Parladé, Massimo Castellari, Fermín I. Milagro, Carlos J. González-Navarro

**Affiliations:** 1 Center for Nutrition Research, University of Navarra, 31008 Pamplona, Spain; paranaz@unav.es (P.A.); apenafuente@alumni.unav.es (A.P.); fmilagro@unav.es (F.I.M.); 2 Navarra Institute for Health Research (IdiSNA), 31008 Pamplona, Spain; 3 Department of Pharmacology and Toxicology, MITOX Research Group, School of Pharmacy and Nutrition, University of Navarra, 31008 Pamplona, Spain; avettora@unav.es; 4 Food Safety and Preservation Department, Institute of Agrochemistry and Food Technology (IATA-CSIC), 46980 Valencia, Spain; mjfabra@iata.csic.es (M.J.F.); conaba@iata.csic.es (A.M.-A.); amparo.lopez@iata.csic.es (A.L.-R.); 5 Interdisciplinary Platform for Sustainable Plastics towards a Circular Economy—Spanish National Research Council (SusPlast-CSIC), 28006 Madrid, Spain; 6 Institute of Agriculture and Food Research and Technology (IRTA), Cabrils Centre, IRTA, Ctra. Cabrils Km. 2, 08348 Barcelona, Spain; Joan.Pera@irta.cat (J.P.); xavier.parlade@irta.cat (J.P.); 7 Institute of Agriculture and Food Research and Technology (IRTA), Finca Camps i Armet s/n, 17121 Monells, Spain; massimo.castellari@irta.cat; 8 Centro de Investigación Biomédica en Red de la Fisiopatología de la Obesidad y Nutrición (CIBERObn), Instituto de Salud Carlos III, 28029 Madrid, Spain

**Keywords:** bioactive compounds, nutraceutical fungi, obesity, metabolic syndrome, insulin, food ingredients

## Abstract

In recent years, food ingredients rich in bioactive compounds have emerged as candidates to prevent excess adiposity and other metabolic complications characteristic of obesity, such as low-grade inflammation and oxidative status. Among them, fungi have gained popularity for their high polysaccharide content and other bioactive components with beneficial activities. Here, we use the *C. elegans* model to investigate the potential activities of a *Grifola frondosa* extract (GE), together with the underlying mechanisms of action. Our study revealed that GE represents an important source of polysaccharides and phenolic compounds with in vitro antioxidant activity. Treatment with our GE extract, which was found to be nongenotoxic through a *SOS/umu* test, significantly reduced the fat content of *C. elegans*, decreased the production of intracellular ROS and aging–lipofuscin pigment, and increased the lifespan of nematodes. Gene expression and mutant analyses demonstrated that the in vivo anti-obesity and antioxidant activities of GE were mediated through the *daf-2/daf-16* and *skn-1/nrf-2* signalling pathways, respectively. Taken together, our results suggest that our GE extract could be considered a potential functional ingredient for the prevention of obesity-related disturbances.

## 1. Introduction

Obesity and other metabolic syndrome-related diseases, including type 2 diabetes, cardiovascular disease, and hypertension, are dramatically increasing worldwide, contributing to significantly increased healthcare spending in every country [1]. Moreover, the high body mass index and adiposity characteristic of obesity can lead to other metabolic and physiological alterations, such as low-grade inflammation, oxidative stress, or premature aging [2,3].

In recent years, efforts have been made to develop different strategies to reduce excess adiposity in obese patients, but also to prevent the appearance and progression of other concomitant metabolic complications [1]. Thus, different groups have focused their efforts on the identification of bioactive compounds found in different food ingredients whose incorporation in the diet might exert beneficial properties in terms of the prevention and/or treatment of these obesity-related disturbances [4]. In this context, numerous studies have reported the antioxidant, anti-obesity, and anti-aging activities of different bioactive compounds (BACs), including phenolic compounds and poly- and monounsaturated fatty acids [5,6,7]. In this sense, different in vitro and in vivo models have been used to screen the ability of these BACs to regulate lipid and carbohydrate metabolism, together with the characterization of the mechanisms underlying these effects [8,9,10].

Recently, mushrooms have emerged as an important source of bioactive compounds with different potential biological properties, including antioxidant, anti-inflammatory, antidiabetic, and lipid-modulating activities, which has led to their consideration as interesting candidates in obesity and other metabolic syndrome-related diseases [11]. Among them, *Grifola frondose* is an edible mushroom of the family Polyporeceae, widely used and consumed in Asia as a traditional food [12]. *G. frondose* is considered a functional food [13] with different health-promoting properties due to its high content in bioactive compounds with antitumoral, antioxidant, and anti-inflammatory activity [14]. Most of the health-promoting activities are attributed to its content in functional polysaccharides such as β-glucans [14,15], which have been demonstrated to exert antidiabetic properties in both cellular and in vivo models [16]. Thus, different works have reported that the consumption of *G. frondosa*’s body, as well as polysaccharides from this mushroom, exert hypoglycaemic and hypolipidemic effects in high-fat diet (HFD)- and streptozocin (STZ)-induced diabetic rodents [17,18,19,20]. Moreover, this mushroom contains other bioactive molecules, including phenolic compounds, ascorbic acid, α-tocopherol, and flavonoids with antioxidant properties [12,21].

Although some studies have proposed anti-obesity properties of *G. frondosa*, little is known about the potential application of this mushroom in the prevention of the complications characteristic of obesity, such as excess adiposity and pro-inflammatory status, together with the potential molecular mechanisms underlying these effects.

In this work, we evaluate the anti-obesity, antioxidant, and anti-aging activities of a *Grifola frondosa* extract (GE) rich in phenolic compounds, using an in vivo model of *C. elegans*. The potential genotoxic activity of this GE extract has also been evaluated though a SOS/umu test, together with an investigation of the involvement of the *skn-1/nrf-2* and *daf-2/daf-16* signalling pathways in the health properties found for this extract.

## 2. Materials and Methods

### 2.1. Grifola Frondosa Fruit Body Production

The control substrate, a standard substrate for the commercial production of edible mushrooms, consisted of 1500 mL chestnut wood chips, 1000 mL chestnut sawdust, and 92.6 g cereal seeds (equal parts corn, barley, and wheat), adjusted to 60% humidity and with a pH of 5.5–6. Polypropylene bags (resistant to high temperatures) were used for cultivation, with ventilation windows fitted with filters that allow the exchange of gases but prevent the loss of moisture or external contamination (Sac O_2_^®^, Deinze, Belgium). All culture bags were filled with a final 4 L quantity of substrate and autoclaved at 100 °C for 2 h.

For the inoculation of the culture bags, once disinfected, a commercial inoculum of *Grifola frondosa* M9827 from MYCELIA BVBA^®^ (Deinze, Belgium; https://www.mycelia.be/en (accessed on 26 January 2019)) was used. The inoculation dose used was 1:30 (inoculum/substrate, *v*/*v*) to shorten incubation times. Inoculum viability was verified by plating an aliquot on MEA (malt extract agar) plates. The inoculated bags were incubated at 22–25 °C for eight weeks. After the incubation period, once the mycelium had grown into the entire volume of the substrate and the absence of contaminants was visually verified, the culture bags were transferred to flowering (or fruiting) rooms to induce mushroom production. To do this, the bags were opened, and the environmental conditions were modified, reducing the temperature to 15 ± 3 °C and increasing the relative humidity to 80–90%. The cultures were kept under these conditions for two weeks and the mushrooms produced were harvested.

### 2.2. Grifola Frondosa Extract Preparation and Composition

The fruiting bodies were freeze-dried, ground, and kept dry at room temperature before extraction. Based on screening tests, the extraction was made with a solid–liquid ratio of 1:20 (*w*/*v*) in hot water (70 °C) on a hotplate with magnetic stirring for 3 h. After that, the GE-based solution was first filtered with a muslin cloth and precipitated by addition of 96% (*v*/*v*) ethanol at a ratio of 1:4 (*v*/*v*). The coagulated GE was centrifuged and freeze-dried for further analysis.

The GE protein content was evaluated, in triplicate, based on the nitrogen content estimated with the Kjeldahl method (BÜCHI K350, Flawil, Switzerland), multiplied by a factor of 6.25 (AOAC, 1998). The monosaccharide composition was determined, in triplicate, by acid hydrolysis followed by chromatographic analysis [22]. The samples were hydrolysed with 2 M trifluoroacetic acid (TFA) at 121 °C for 3 h, subsequently dried under a stream of air, and dissolved in distilled H_2_O. The hydrolysed monosaccharides were analysed using high-performance anion exchange chromatography with pulsed amperometric detection (HPAEC-PAD) using an ICS-3000 system (Dionex, Sunnyvale, CA, USA) equipped with a CarboPac PA1 column (4 × 250 mm, Dionex).

The ash content was determined by calcination (AACC 08-01, 2000). Samples were placed in a crucible and heated in a muffle oven at 550 °C for 12 h. Then, the weight of the crucible was recorded, and the ash content was calculated by weight difference.

Total phenolic content was estimated by the Folin–Ciocalteu colorimetric assay [23]. Briefly, the Folin–Ciocalteau reagent was mixed with different concentrations of the samples, 0.4 mL of sodium carbonate (16%) was added, and the samples were incubated for 30 min at room temperature before reading the absorbance values at 765 nm. A calibration curve was built using gallic acid as a standard, and the total phenolic content was expressed as gallic acid equivalent (mg) per 100 g of the dry weight of the extract (mg GAE/100 g dry weight). All determinations were carried out in triplicate.

Moisture (90.87%) was assessed gravimetrically by measuring the weight loss after drying at 105 °C in a convection oven.

### 2.3. Antioxidant Capacity of the GE Extract

The Trolox Equivalent Antioxidant Capacity (TEAC) of the GE was determined using a modification of the original TEAC method [24]. Trolox (6-hydroxy-2,5,7,8-tetramethylchroman-2-carboxylic acid) was used as a standard for antioxidant capacity. Samples were dissolved in distilled water for 12 h and analysed for ABTS+·(2,2-azinobis (3-4ethylbenzothiazoline)-6-sulfonic acid) radical scavenging activity. First, the ABTS+·solution with an initial absorbance at 734 nm of 0.70 ± 0.08 was prepared, then 20 μL of each GE solution was added to 230 μL of the ABTS+ solution and the absorbance was registered at 6 min. For calibration, Trolox standards of different concentrations (0.02–0.30 μmol mL^−1^) were prepared, and the same procedure was followed. The TEAC of the GE samples was determined by comparing the corresponding percentage of absorbance reduction at 6 min with the Trolox concentration–response curve. All the determinations were carried out at least six times using a spectrophotometer (CLARIOstar, BMG LABTECH, Ortenberg, Germany) with water as the blank.

### 2.4. Genotoxicity Screening Assay (SOS/umu Test)

#### 2.4.1. Exposure Concentrations and Solubility Test

For the SOS/umu test, GE was dissolved in water at 40 mg/mL. This concentration was selected based on the extract’s solubility in water (the extract showed some precipitation when prepared at 80 mg/mL). The final concentration in the 96-well plates (plate B, see Section 2.4.2) was 1 mg/mL (1/40 dilution).

The positive control stock solutions were prepared in DMSO at 0.5 mg/mL (corresponding to 0.0125 mg/mL in 96-well plate B) for 2-aminoanthracene (2-AA) (Sigma-Aldrich, Taufkirchen, Germany) and at 100 µg/mL (2.5 µg/mL in 96 well-plate B) for 4-nitroquinoline-*n*-oxide (4-NQO) (Sigma-Aldrich, Beijing, China). 4NQO was the positive control without metabolic activation (PBS) and 2AA with metabolic activation (S9). Water was used as the negative control.

#### 2.4.2. SOS/umu Test

An SOS/umu test was used to determine the DNA-damaging effect of GE and was carried out according to the method of Oda et al. [25] and Reifferscheid et al. [26] with some modifications. The test strain *S. typhimurium* TA1535/pSK1002 (German Collection for microorganisms and cell cultures (DSMZ)) from stock (−80 °C; in TGA medium containing 10% DMSO as cryoprotective agent) was thawed and 0.5 mL of bacteria were suspended in 100 mL TGA medium supplemented with ampicillin (50 µg/mL). The tester strain suspension was incubated overnight at 37 °C with slight orbital shaking (155 rpm) until an optical density was reached (OD 600 between 0.5 and 0.8). Thereafter, the overnight culture was diluted with fresh (not supplemented with ampicillin) TGA medium and incubated for 2 h at 37 °C and 155 rpm in order to obtain log-phase bacteria exponential growth culture (OD 600 between 0.20 and 0.35). The test was performed in the presence and absence of an external metabolic activation system (10% of rat S9 mix, prepared from S9 SD rat liver Aroclor KCl frozen, Trinova, Germany) in order to determine the possible genotoxic effects of any metabolite. For each test performed, negative and positive controls were included, water was used as solvent control (negative control), and 4-nitroquinoline-N-oxide (4-NQO) (Sigma-Aldrich, China) and 2-aminoanthracene (2-AA) (Sigma-Aldrich, Germany) were used as positive controls in the absence and presence of S9 mix, respectively (see the maximal concentrations used in Section 2.4.1).

The test procedure followed was as follows: initially, GE and positive controls were dissolved at their maximum concentrations (see Section 2.4.1). Subsequently, 20 µL was placed in a 96-well plate and 10 serial half-dilutions in DMSO (positive controls) or water (*Grifola frondosa* extract) were prepared in a 96-well plate (plate A). The final volume in each well was 10 µL. The negative control (water) was placed in the wells of the last row of the plate. Then, 70 µL water was added to each well. The absence of any precipitation of the extract was checked for at this point. Thereafter, in two more 96-well plates (plate B; one for the test with S9 and the other without S9), 10 µL S9 mix or 10 µL PBS, respectively, were added followed by 25 µL of each concentration of GE or a positive control, with concentrations previously prepared. Finally, 90 µL of exponentially growing bacteria was added to each well and both plates were incubated during 4 h by shaking (500 rpm) at 37 °C. After the incubation period, the absorbance at 600 nm was measured in order to evaluate the toxicity on *S. typhimurium* TA1535/pSK1002 (two B plates).

We calculated the toxicity as follows:

(1) $$\%\;\mathrm{Survival}=\frac{A_{600}\;\mathrm{for}\;\mathrm{each}\;\mathrm{concentration}\;\mathrm{tested}\;}{\mathrm{Average}\;A_{600}\;\mathrm{for}\;\mathrm{negative}\;\mathrm{control}\;}\;\text{×}\;100.$$

Then, for the determination of β-galactosidase activity, 30 µL/well of treatment plates (plate B) were transferred into two new 96-well plates (plates C) containing 150 µL/well of ONPG solution (2-nitrophenyl-β-D-galactopyranoside, Sigma-Aldrich, Schaffhausen, Switzerland): 0.9 mg/mL in B-buffer was prepared according to Reifferscheid et al. [26] for an enzymatic reaction. C plates were incubated for 30 min at 28 °C with orbital shaking (500 rpm) in the dark. After the incubation period, 120 µL of Na_2_CO_3_ (1 M) were added per well to stop the reaction. The absorbance (A_420_) was measured, and β-galactosidase activity was determined as follows (two C plates):

β-galactosidase activity relative units (RU):

(2) $$\mathrm{RU}\;:=\frac{A_{420}\;\mathrm{for}\;\mathrm{each}\;\mathrm{concentration}\;\mathrm{tested}\;}{\;A_{600}\;\mathrm{for}\;\mathrm{each}\;\mathrm{concentration}\;\mathrm{tested}\;}.$$

Additionally, induction factor (IF)

(3) $$\mathrm{IF}=\frac{\mathrm{RU}\;\mathrm{for}\;\mathrm{each}\;\mathrm{concentration}\;\mathrm{tested}\;}{\;\mathrm{Average}\;\mathrm{RU}\;\mathrm{for}\;\mathrm{negative}\;\mathrm{control}\;},$$

where the average β-galactosidase RU for the negative control was as follows:

(4) $$\mathrm{RU}\;:=\frac{\;\mathrm{Average}\;A_{420}\;\mathrm{for}\;\mathrm{negative}\;\mathrm{control}\;}{\;\mathrm{Average}\;A_{600}\;\mathrm{for}\;\mathrm{negative}\;\mathrm{control}\;}$$

Correspondingly, β-galactosidase relative units were calculated for both positive controls. Under the given test conditions, only when the positive controls reached an IF ≥ 2, the test was considered valid. Finally, the extract is considered genotoxic when the induction factor is ≥2 at nontoxic concentrations (bacteria survival percentage ≥ 80%) in any of the conditions studied (with or without metabolic activation). The analysis excluded those wells where precipitation was observed.

### 2.5. C. elegans Strains, Culture, and GE Treatment

Wild-type N2 Bristol strain and *daf-16* (mu86, CF1038) and *skn-1* (mg570, GR2245) mutants were purchased from the Caenorhabditis Genetics Center (CGC, University of Minnesota, MN, USA). All strains were grown at 20 °C on a Nematode Growth Medium (NGM) with *Escherichia coli* OP50 as the normal nematode diet.

*C. elegans* was cultured as previously described [27,28]. Experiments were conducted using six-well cell culture plates with 4 mL of NGM per well, with four replicates for each condition. As a fat reduction control, Orlistat-supplemented plates (6 µg/mL Orlistat; Sigma-Aldrich, St. Louis, MO, USA) were used [27,29]. The media was supplemented as previously described in [30], with some modifications. *Grifola frondosa* extracts were dissolved in ultrapure water and tested at concentrations of 10 and 20 µg/mL. The same amount of water was added to non-supplemented plates as a control. The plates were protected from light oxidation by maintenance in a dark environment during solidification of the agar. Subsequently, an overnight culture of *E. coli* OP50 was seeded (200 µL/well) and plates were incubated at room temperature in darkness until dry [27].

For all procedures, standard hypochlorite treatment of gravid animals was used to obtain age-synchronized L4 adult worms. The eggs were allowed to hatch overnight in M9 medium and about 750 L1 individuals (larval stage) were added per well onto supplemented plates. After 46 h, nematodes reached the L4 stage (adult), when worms were collected, and assays were performed.

### 2.6. Nile Red and Oil Red O (ORO) Staining Methods

Nile Red (a dye for neutral lipids—#N3013, Sigma-Aldrich, USA) staining was performed as previously described [27,28,30], with minor modifications. Briefly, L4 worms were harvested in 1.5 mL tubes and washed two times with PBST (0.01% of Triton X-100 in phosphate-buffered saline). Then, worms were maintained for 15 min on ice to stop pharyngeal pumping and fixed in 40% isopropanol for 3 min. Then, fixed worms were stained by adding 150 µL of Nile Red solution (3 µg/mL) and incubated for 30 min at 20 °C in the dark with gentle rocking. After this time, worms were washed in PBST and mounted on a 2% agarose pad for microscopy visualization.

ORO staining was performed as previously described [30,31]. The day before the staining of the worms, a fresh ORO solution was prepared by diluting stock (0.5% ORO in isopropanol) to a 60% solution with water, filtering (0.45 µM filter), stirring at room temperature overnight, and filtering again just before use. Afterwards, L4 worms were collected, washed, and fixed in 60% isopropanol for 5 min. Then, the fixed worms were incubated in the ORO solution for 6 h in a wet chamber with gentle shaking in the dark, washed with PBS, and mounted on a 2% agarose pad for microscopy visualization.

### 2.7. DHE Staining

The fluorescent dye dihydroethidium (DHE; Dihydroethidium BioReagent, ≥95% (HPCE), Sigma-Aldrich, USA) was used to measure the in vivo levels of ROS as previously described [30,32,33]. Briefly, synchronized 500 L1 larvae were transferred onto plates containing water (control) or GE (10 and 20 µg/mL), and were grown until the L4 stage, when worms were collected. After washing in PBS three times, worms were kept in a 3 µM DHE solution (in PBS) for 30 min. After this time, worms were washed and mounted on 2% agarose pads with 1% sodium azide.

### 2.8. C. elegans Aging Visualization

Synchronized 500 L1 larvae were transferred onto plates containing water (control) or GE (20 µg/mL) and grown until the L4 stage. Worms were collected, washed, and mounted onto 2% agarose pads with 1% sodium azide. The auto-fluorescence of the worm attributed to the lipofuscin pigment was quantified as a marker of aging [34].

### 2.9. Heat Stress Resistance Assay

Synchronized 500 L1 larvae were placed onto plates containing water (control) or GE (20 µg/mL) and grown until the L4 stage. Four replicates were used for each condition. At that point, plates containing the L4 worms were transferred to an incubator at 35 °C [35]. Dead and alive worms were recorded every 2 h. The absence of response after a gentle touch with a platinum wire indicated the death of the worms.

### 2.10. Image Acquisition and Quantification

Approximately 300 animals were fixed and stained for all conditions tested. Fluorescent images of Nile Red-stained worms were captured at 100× magnification on a Nikon SMZ18 research stereomicroscope equipped with an epi-fluorescence system and a DS-FI1C refrigerated colour digital camera (Nikon Instruments, Inc., Tokyo, Japan). Images were taken with the same conditions and integration time under a GFP filter (Ex 480–500; DM 505; BA 535–550). For the ORO analysis, images were also captured at 100× magnification with a Nikon SMZ18 research stereomicroscope equipped with a Nikon DS-Fi2 high-definition colour camera. The auto-fluorescence of the lipofuscin pigment and the fluorescence intensity of the DHE-labelled ROS formation were measured with a Nikon Eclipse 80i epi-fluorescent microscope, equipped with DAPI (with excitation at 340–380 nm and emission at 435–485 nm) and TRITC (Ex 540–625; DM 565; BA 605–655) filters, respectively. All the image analyses were performed with the ImageJ software (National Institutes of Health, NIH, MD, USA), as previously described [27]. The mean value (fluorescence mean value per pixel), the integrated density, and the volume of the worms were determined. Approximately 25–40 worms were examined for each replicate, using four replicates per condition in three independent experiments.

### 2.11. Lifespan Assay

For the lifespan analyses, synchronized L1 larvae were transferred to NGM plates containing water (control group) or GE (10 and 20 µg/mL) for 46 h at 20 °C to allow *C. elegans* to develop to the L4 stage. Four replicates were used per condition in two independent experiments. After this time, 50 to 65 L4 larvae per replicate were then transferred onto new plates containing 40 µM of 5-Fluoro-2-deoxyuridine (FUDR, #856657, Sigma-Aldrich, USA), together with the additional supplement (water or GE for the control and the treated groups, respectively). Surviving and dead animals were counted daily, until all nematodes died. The absence of response after a gentle touch with a platinum wire indicated the death of the worms.

### 2.12. Egg Laying and Worm Size

The egg laying of young adult nematodes (day 3 of growth) was compared between worms grown on GE supplemented or not-supplemented NGM agar plates. The images were taken at 135× magnification using a Nikon SMZ18 stereomicroscope equipped with a Nikon DS-Fi1C high-definition colour camera. Worm length (µM) and area (µM^2^) were calculated with Nikon NIS-ELEMENTS Software.

### 2.13. RNA Extraction and Quantitative PCR Analyses

Total RNA from *C. elegans* N2 strain were extracted using a Trizol^®^ RNA isolation reagent (Thermo Fisher Scientific, Paisley, UK) following the manufacturer’s instructions. Absorbance at 260/280 nm in a NanoDrop ND-1000 spectrophotometer (Thermo Fisher Scientific, Wilmington, DE, USA) was used to determine the concentration and purity of RNA. DNA-free RNA was obtained for all samples by treating 1000 ng of RNA with DNAse (Ambion™ DNase I, RNase-free; Thermo Fisher Scientific, Inc., Waltham, MA, USA) following the manufacturer’s instructions and then reverse-transcribed into cDNA following the previously described protocol [30].

Gene expression analyses were performed by quantitative real-time PCR (qPCR) using the CFX384 Touch™ Real-Time PCR Detection System (BioRad, Hercules, CA, USA). For these assays, TaqMan Universal PCR master mix and specific probes (Table S1) from Applied Biosystems Technologies (Thermo Fisher Scientific, Inc., Waltham, MA, USA) and Integrated DNA Technologies, Inc. (Coralville, IA, USA) were used. The expression level of *pmp-3* gene was used as housekeeping control gene [36]. Gene expression differences between treated and untreated worms were quantified using the relative quantification 2^−∆∆Ct^ method [37].

### 2.14. Statistical Analyses

Data from Nile Red, Oil Red O, oxidative stress (DHE) and lipofuscin determinations were evaluated by a hierarchical ANOVA test, where replicates were nested in treatments, followed by multiple comparison (Fisher’s protected Least Significant Difference, LSD) tests. Log-rank (Mantel–Cox test) between GE-treated and control (NGM) groups were performed for lifespan analyses. Wilcoxon test was used for comparing each treatment to its control in the real-time PCR analyses. All tests were performed using StataSE v12 software (StataCorp, LLC, College Station, TX, USA).

## 3. Results and Discussion

Mushrooms are widely consumed foods whose high contents of bioactive compounds may provide antioxidant, anti-inflammatory, anti-obesity, and antidiabetic properties, among others [38]. Thus, different in vitro and in vivo models have been used to identify and characterized the health benefits of these mushrooms for their potential application as food ingredients. Thereby, *Grifola frondosa* polysaccharides have been found to exert antioxidant and antidiabetic properties in different animal models [14,16,17,39,40].

The regulatory pathways of energy homeostasis are highly conserved between *C. elegans* and mammals, making this nematode a powerful model for exploring the genetic bases of fatty acid synthesis and the regulation of fat storage. Thus, *C. elegans* has been widely used as a screening model for the identification and evaluation of BACs with healthy properties in the prevention of obesity-related disturbances, together with the characterization of the biological mechanism underlying these effects. Moreover, this nematode has been widely used for determining the antioxidant, anti-aging, and life-prolonging properties of BACs present in different food ingredients with beneficial properties in the prevention of aging-related diseases [41,42].

### 3.1. Grifola Frondosa Extract (GE) Composition and In Vitro Antioxidant Capacity

The composition and detailed monosaccharide and phenolic compound contents of the *Grifola frondosa* extract (GE) obtained through the extraction process described in Section 2.1 are compiled in Table 1. The extraction yield was around 6.62 ± 0.48 and GE was mainly composed of carbohydrates and proteins, but also contained significant amounts of phenolic compounds and ashes. The carbohydrate analysis revealed the presence of significant amounts of glucose, which can be ascribed to a high β-glucan content (around 20%) in the GE, similar to other *G. frondosa* extracts previously described [38]. Smaller amounts of mannose, fucose, and galactose may be ascribed to typical fungal mannogalactans and fucogalactans.

The total polyphenolic content was in the same range as other *Grifola* water extracts with antioxidant properties reported [12]. The phenolics content was also similar to other mushroom extracts obtained from *Craterellus curnicopioides* and *Hydnum repandum* [43], but lower than that found for *Lentinula edodes* and *Pleorotus osteatus* [44]. The presence of polyphenols was directly related to the antioxidant capacity of the extract, according to the results reported for this mushroom [12] and for other species, such as *Pleurotus ostreatus*, *Agaricus bisporus*, *Ganoderma Lucidum*, and *L. edodes* [45], known for their high antioxidant activities. Thus, our data show that the GE extract represents an important source of β-glucans and phenolic compounds with in vitro antioxidant activity.

### 3.2. The GE Extract Did Not Show Genotoxic Activity

Prior to the in vivo evaluation of the GE extract, a preliminary screening of its potential genotoxicity was evaluated by a SOS/umu test. Although this test is used for screening purposes, a high degree of agreement has been found with the standardized Ames test (OECD guideline 471) for mutagenicity testing [46,47].

**Figure 1:**
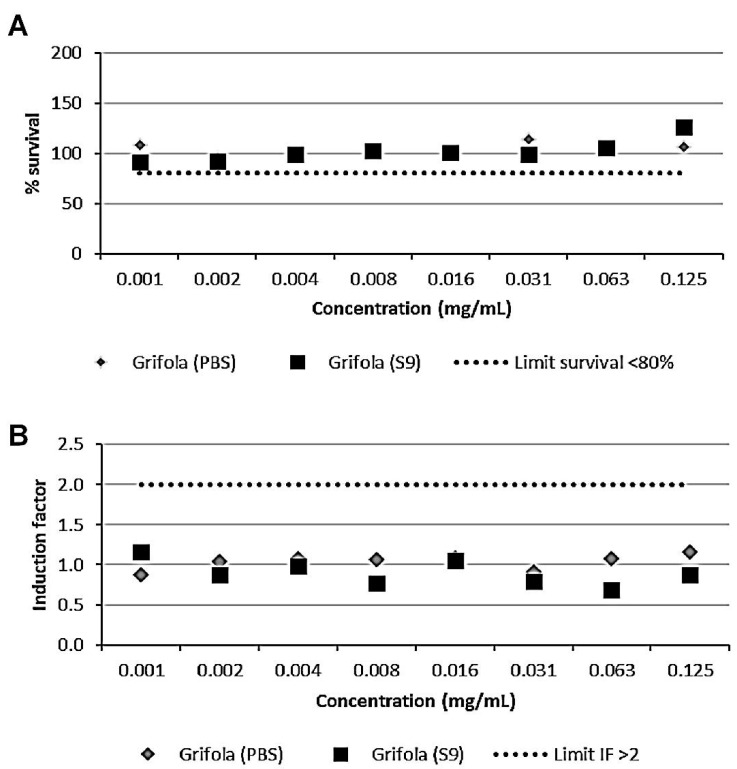
Results from SOS/umu test with (black) or without (grey) S9 activation. (**A**) Bacterial survival is shown as a percentage. Concentrations are considered nontoxic if survival is >80%. (**B**) Genotoxicity. A compound is considered genotoxic if the induction factor is ≥2 at nontoxic concentrations for the bacteria in any of the conditions tested.

All the controls used for the SOS/umu test were correct (IF < 2 for negative and IF > 2 for positive controls). The wells corresponding to the higher concentrations tested (final concentrations in the wells: 1, 0.5, and 0.25 mg/mL) showed precipitation in the wells and were discarded from the analysis. According to the results (Figure 1), GE was considered nongenotoxic as the induction factor was always lower than 2 at nontoxic concentrations in the absence or presence of metabolic activation (S9 fraction).

### 3.3. The GE Extract Reduced C. elegans Fat Accumulation without Affecting Worm Development

Previous studies have suggested the anti-obesity properties of *G. frondosa*. For example, Aoki and colleagues demonstrated that supplementation with 0.4% *G. frondosa* extract in high fat-induced obese mice for 15 weeks significantly reduced body weight gain and visceral fat accumulation, ameliorated hepatic triglyceride storage, and improved glucose tolerance [48]. They suggested that the anti-obesity and antidiabetic properties of this *G. frondosa* extract were attributed to its activity as a PPARδ agonist.

As mentioned above, our GE represents an important source of different BACs, including beta-glucans, phenolic compounds, PUFAs, and MUFAs. Different studies have reported the lipid-reducing activity of different flavonoids and phenolic acids in *C. elegans*, including ours [30]. For this reason, we aimed to determine if the combination of the bioactive compounds found in our GE could also affect the lipid homeostasis using the *C. elegans* model. The intestinal and hypodermal cells of this nematode accumulates lipids in the form of fat droplets, which can be detectable under microscopy using fat-soluble dyes, such as Sudan Black B, Oil red O, and Nile Red [49,50]. The quantification of the fluorescence of the fixative Nile Red lipophilic dye has been demonstrated to represent a reliable method to determine the fat content of this nematode, and has been widely used for evaluating the lipid-reducing activity of BACs, with potential uses in the prevention of and therapy for obesity-related diseases [27,30,41,51].

**Figure 2:**
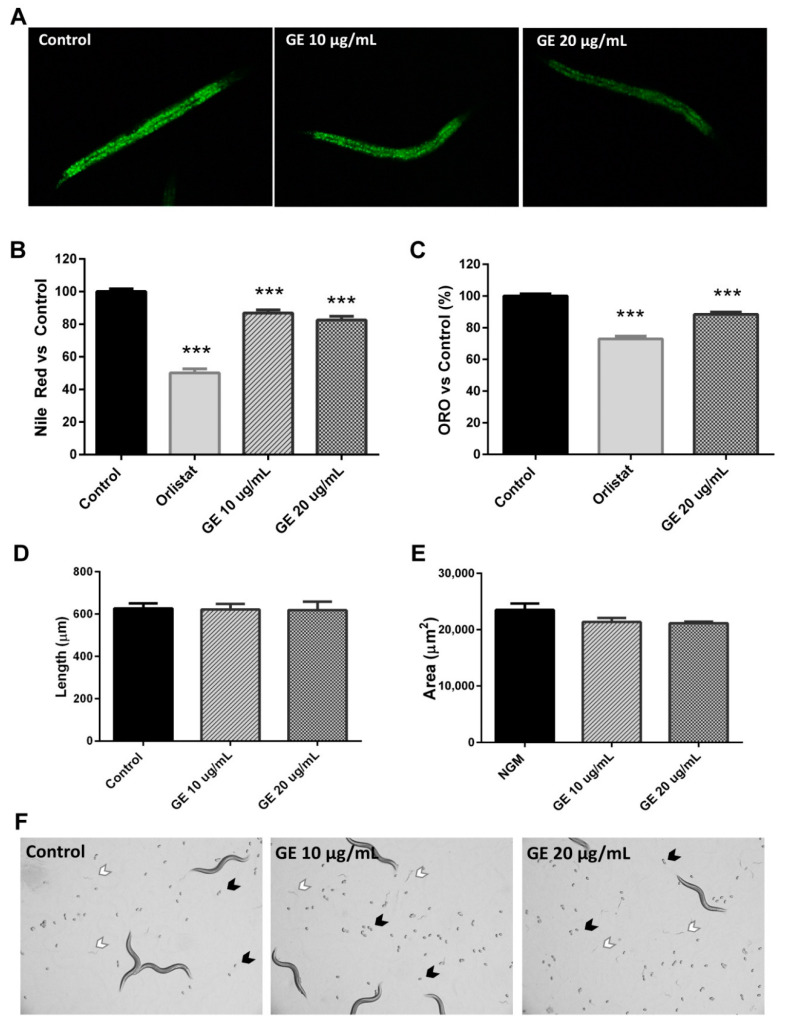
*Grifola frondosa* extract (GE) reduces the fat content of *C. elegans* from L1 to L4 independently of the effect on worm development. (**A**) Microscopic visualization of the worm fat content of control and GE (10 and 20 µg/mL)-treated worms after staining with Nile Red. (**B**) Nile Red staining quantification of control and GE-treated (10 and 20 µg/mL) worms. Orlistat (6 µg/mL) was used as the positive control. (**C**) Oil Red O staining quantification of control and GE-treated worms (20 µg/mL). (**D**) Length (µM) of GE-treated and untreated worms on day 1 of adulthood. (**E**) Area (µM^2^) of GE-treated and untreated worms on day 1 of adulthood. All results are expressed as the mean ± SEM relative to NGM control worms. Significance refers to the effect of the treatments with respect to NGM control worms (ANOVA followed by LSD test, *** *p* < 0.001). (**F**) Microscope observation of the presence of eggs (black arrows) and L1 larvae (white arrows) in control (NGM) and GE-supplemented plates.

For this experiment, L1 N2 wild-type worms were treated until reaching the L4 stage with and without the GE at the doses of 10 and 20 µg/mL, when nematodes were collected, fixed, and stained with Nile Red (Figure 2A). As revealed by the quantification of the fluorescence of the worms (main value per pixel), both doses of GE induced a significant reduction in the lipid content of *C. elegans*, in comparison with untreated control worms (Figure 2B). Orlistat-treated worms were used as a positive control of fat reduction. In fact, the reduction induced by the high dose of GE was 18.64%, and a similar result was obtained after Oil Red O staining (Figure 2C), confirming this effect on worm fat deposition.

Although our extract did not exhibit in vitro genotoxicity in the SOS/umu test, these results could be related to an effect on nematode development. Thus, in order to dismiss this negative effect, we analysed the effect of GE extract treatment from L1 to day 1 of adulthood (72 h of treatment) on worm length, size, and egg laying. No differences were observed in terms of worm length (Figure 2D) and size (Figure 2E) between GE-treated and untreated nematodes, suggesting that the lipid-reducing activity of GE is not accompanied by an effect on worm length and size. Furthermore, after 72 h of treatment, both GE and control plates exhibited the presence of both eggs (Figure 2F, black arrows) and L1 larvae (Figure 2F, white arrows) without differences in the time of appearance. All these results suggest that treatment with our GE from L1 to L4 significantly reduces the *C. elegans* fat content independently of any effect on worm development.

### 3.4. The Fat-Reducing Activity of GE in C. elegans Is Mediated by the DAF-16/FOXO Signalling Pathway

In order to deepen our knowledge of the underlying mechanisms of the anti-obesity activity of GE seen in *C. elegans*, we quantified the expression of different genes involved in both lipid and carbohydrate metabolism. Thus, we evaluated the expression of key lipogenic (*fat-5*, *fat-7*, *elo-5*, *elo-6*) and β-oxidation genes (*acox-1*, *maoc-1*, *daf-22*), together with the insulin-pathway mediators (*daf-2*, *daf-16*). Due to the high carbohydrate, phenolic, and FA content previously observed in our GE, we also analysed the expression of *skn-1*, an ortholog of the human *NRF-2* gene, and *sod-3*, involved in oxidative stress and longevity.

**Figure 3:**
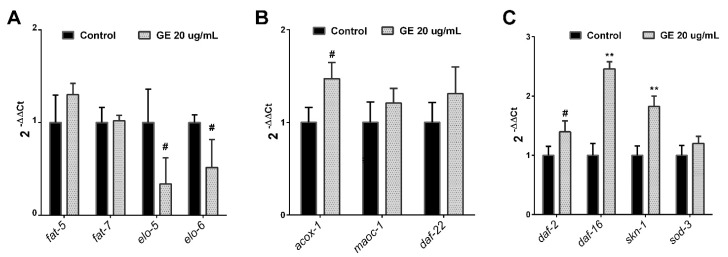
Gene expression levels of lipogenesis-related genes (**A**), β-oxidation-related genes (**B**), and daf-2/daf-16 and skn-1/nrf-2 signalling pathways (**C**). Results are expressed as the fold difference expression levels of each gene in GE-treated worms compared with the control, calculated with the 2^-∆∆Ct^ method. Significance refers to the effect of GE with respect to untreated control worms (ANOVA followed by LSD comparisons, # *p* < 0.1; ** *p* < 0.01).

No differences were observed in the lipogenesis-related genes *fat-5* and *fat-7* (*p* > 0.05) between GE-treated (20 µg/mL) and untreated worms (Figure 3A). However, GE-treated worms exhibited a tendency to downregulate two fatty acid elongases (elo-5 and elo-6) involved in the synthesis of long-chain fatty acids [52], which suggests a potential inhibitory activity of GE on lipid biosynthesis. Moreover, although no differences were observed in *maoc-1* and *daf-22* genes, GE-treated worms also showed a tendency to acox-1 overexpression (Figure 3B), with an ortholog of human ACOX-1 (acyl-CoA oxidase 1) involved in the first step of the peroxisomal beta-oxidation of long-chain fatty acids [53]. The GE-induced overexpression of acox-1, together with the reduced lipid biosynthesis, would suggest that the fat-reducing activity could be partially mediated by a significant increase in the peroxisomal breakdown of long-chain fatty acids.

Importantly, the effect of GE on fatty acid synthesis and breakdown was also accompanied by a tendency to higher expression of *daf-2* (Figure 3C), an ortholog of human IGF1R (insulin like growth factor 1 receptor), INSR (insulin receptor), and INSRR (insulin receptor-related receptor). This gene codes for the single receptor protein in the IIS pathway. Moreover, treatment with GE induced a pronounced overexpression of daf-16, the ortholog of human FOXO, which codes for a key transcription factor regulated by the IIS pathway. Daf-16 acts as a nutrient-sensing regulator of energy homeostasis and lipid metabolism [52,54]. The significant overexpression of GE on daf-16/foxo was also confirmed at a lower dose of the extract (10 µg/mL) and would suggest that the anti-obesity properties previously observed with GE are mediated by the upregulation of this transcription factor.

Finally, GE-treated worms exhibited a significant upregulation of *skn-1* (Figure 3C), an ortholog of the human *NRF2* gene, an important transcription factor of the antioxidant and antiaging responses [55]. No differences were observed in the expression of *sod-3*. Again, significant upregulation of *skn-1* was observed after treatment with a lower dose of GE (10 µg/mL). *SKN-1* activation has been previously shown to be involved in fat metabolism by depleting somatic lipids [56,57], so overexpression of this transcription factor by GE might also be involved in the fat-reducing activity observed in our Nile Red and Oil Red O analyses. Our findings demonstrate that our GE extract reduced the *C. elegans* lipid content when treated from L1 to L4; this effect is mediated by a reduction in the fatty acid biosynthesis and increased oxidation, together with a significant overexpression of the *skn-1* and *daf-16* transcription factors.

In order to further investigate the potential implication of the *daf-2*/*daf-16* and the *skn-1*/*nrf-2* signalling pathways in the anti-obesity properties of the GE extract, we analysed the lipid-reducing activity of GE on *C. elegans* in a glucose-loaded medium. Glucose has been used to establish a *C. elegans* obesity model in various studies [58,59], and has been demonstrated to affect both lipid accumulation and oxidative stress responses [60].

**Figure 4:**
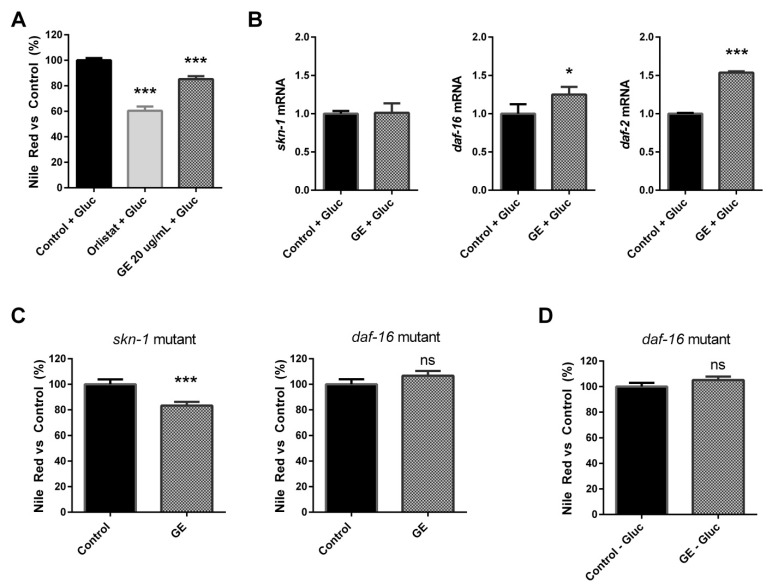
Grifola extract (GE) reduces *C. elegans* fat accumulation in a glucose-loaded medium through *daf-16* and *daf-2* up-regulation. (**A**) Nile Red quantification of NGM-control and GE (20 µg/mL)-treated worms grown in a medium supplemented with glucose (10 mM). Orlistat (6 µg/mL) was used as the positive control. The results are expressed as the mean ± SEM relative to untreated control worms. (**B**) Expression levels of *skn-1*, *daf-16*, and *daf-2* genes in GE-treated worms in comparison with the untreated control worms grown in a medium with glucose overload. Results are expressed as the fold-difference expression levels of each gene in GE-treated worms compared with the control, calculated with the 2^−∆∆Ct^ method. (**C**) Nile Red quantification of NGM control and GE (20 µg/mL)-treated worms for *skn-1* and *daf-16* mutants. (**D**) Nile Red quantification of NGM control and GE (20 µg/mL)-treated *daf-16* mutant worms grown in a medium supplemented with glucose. Significance refers to the effect of the treatments with respect to NGM-control worms (* *p* < 0.05; *** *p* < 0.001).

Again, we observed that treatment with 20 µg/mL of GE induced a significant reduction (14.71%) in the fat content in comparison with untreated control worms in a glucose-loaded (10 mM) medium (LSD *p*-value < 0.001; Figure 4A). A gene expression analysis performed after this assay demonstrated that, when the medium was supplemented with glucose, no differences were observed in *skn-1* gene expression (Figure 4B), suggesting that the *skn-1* activation previously observed might be involved in the potential antioxidant activity of this extract, more than modulating the lipid accumulation in this model. However, the fat-reducing activity of GE in a glucose-loaded medium was mediated by a significant upregulation of the *daf-16* and *daf-2* genes.

DAF-2 and DAF-16 are the most critical components involved in the IIS pathway of *C. elegans*, and constitute one of the major nutrient-sensing pathways that act as regulators of fat metabolism [52,61]. To validate our results, we determined the effect of GE on lipid accumulation using both skn-1 (mg570; GR2245) and daf-16 (mu86; CF1038) mutant strains. As can be observed in Figure 4C, treatment with our GE from L1 to L4 with the skn-1 mutant induced a significant reduction in the lipid content (*p* < 0.001) in comparison with the untreated mutant, demonstrating that this gene would not be crucial for the anti-obesity activity of our extract, which would support our previous findings. However, treatment with the GE extract did not have the effect of lipid accumulation on the daf-16 mutant when grown on NGM plates (Figure 4C), nor in glucose-loaded plates (Figure 4D), suggesting that the activity of this transcription factor is crucial for the lipid-reducing activity of GE. Thus, our findings demonstrate that the fat-reducing activity of our GE extract is dependent on the daf-16/FOXO mediator of the IIS signalling pathway.

### 3.5. The GE Extract Exhibited Antioxidant Activity in C. elegans

An imbalance in the lipid metabolism can lead to an increase in inflammation, which in turn promotes the generation of intracellular reactive oxygen species (ROS), causing oxidative stress. Excessive ROS accumulation can alter proteins and other molecules, such as lipids and DNA, contributing to cellular damage and, in turn, the development of aging-related diseases, including diabetes [62].

Previous studies have suggested the in vivo antioxidant activity of *G. frondosa*, attributed to its content in polysaccharides [63]. Thus, Kou and colleagues suggested that the hypoglycaemic activity of *G. frondosa* polysaccharides seen in diet–streptozotocin-induced diabetic rats were mediated by a reduction in oxidative stress through the NF-kB signalling pathway [39]. Furthermore, other mushrooms have been demonstrated to exert antioxidant activities, some of them evaluated using the *C. elegans* model [64,65].

As we have previously described, our GE exhibited high in vitro antioxidant activity, probably due to its high content in polysaccharides, but also to the presence of phenolic compounds, PUFAs, and MUFAs with antioxidant activities [66,67]. Moreover, as demonstrated before, treatment with GE induced a significant overexpression of skn-1/Nrf, which was not related to the reduced fat content of the worm. This transcription factor plays a critical role in the regulation of the *C. elegans* response to oxidative stress [68], together with additional functions including proteostasis and aging [55].

**Figure 5:**
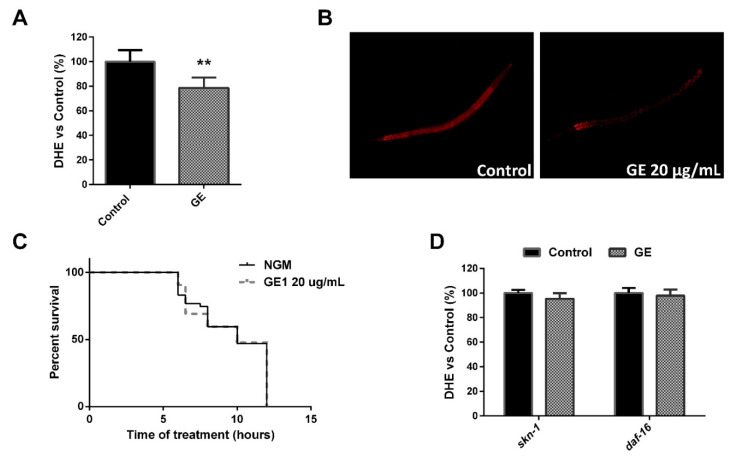
Grifola extract (GE) exhibits in vivo antioxidant activity. (**A**) Microscope detection of the ROS production (stained by DHE) in control and GE-treated (20 µg/mL) worms. (**B**) Quantification of the ROS production (determined by DHE) in GE-treated worms in comparison with untreated control worms (mean ± SD relative to untreated control worms). Significance refers to the effect of GE with respect to untreated control worms (Student’s *t*-test, ** *p* < 0.01). (**C**) Percentage of survival during time (h) of control and GE-treated (20 µg/mL) L4 worms incubated at 35 °C. (**D**) DHE quantification of ROS production in GE-treated worms in comparison with untreated control worms for skn-1 and daf-16 mutants.

The overexpression of daf-16 induced by GE could also result in increased resistance to oxidative stress, in addition to a prolonged lifespan [69]. For this reason, we also evaluated the in vivo antioxidant activity of GE in *C. elegans* when treated from L1 to L4. Thus, treatment with GE (20 µg/mL) induced a significant reduction in ROS production (Figure 5A), quantified by DHE (Figure 5B), suggesting GE’s potential role in reducing oxidative stress in vivo.

One of the methods to determine the oxidative status of the nematodes is to evaluate their tolerance to heat stress. No differences were observed in the percentage of survival between GE-treated and untreated worms exposed to a temperature of 35 °C (Figure 5C), suggesting that the antioxidant activity of GE is independent to any effect on heat stress resistance. However, SKN-1 activity has recently been demonstrated to be suppressed upon heat stress [70]. For this reason, we aimed to further investigate if the ROS-reducing activity of our GE is dependent on skn-1 overexpression. As can be observed, both skn-1 and daf-16 mutants lacked ROS-reducing activity, as no differences were observed between GE-treated and untreated control worms (Figure 5D). Our results demonstrate that GE treatment induces a significant reduction in ROS in *C. elegans* grown under normal conditions, and that this activity is mediated by the skn-1/Nrf-2 and daf-16/FOXO transcription factors.

### 3.6. The Fat-Reducing Activity of GE in C. elegans Is Mediated by the DAF-16/FOXO Signalling Pathway

As mentioned before, SKN-1 and DAF-16 play a critical role in the in vivo antioxidant activity of the *G. frondosa* extract. However, both transcription factors are well-known anti-aging and longevity factors, and their activation is observed in several long-lived models [58,70].

**Figure 6:**
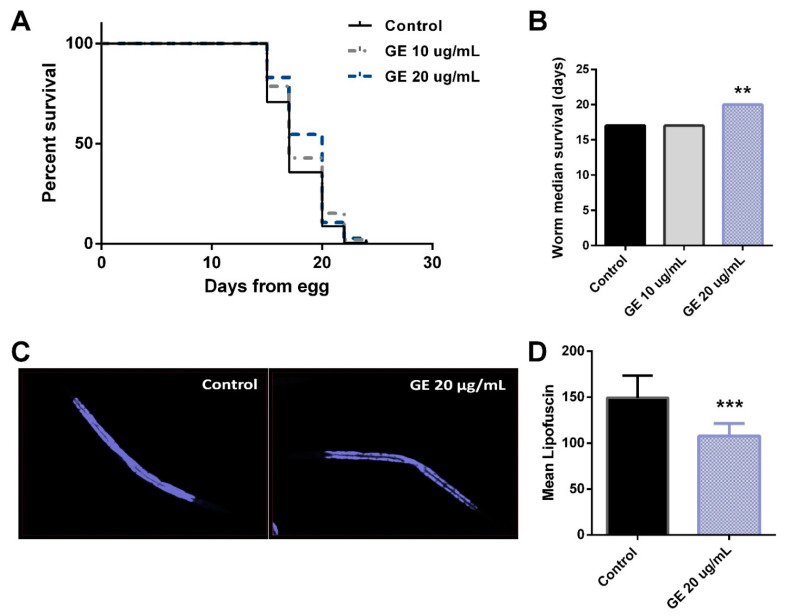
Grifola extract (GE) lengthens the *C. elegans* lifespan. (**A**) Lifespan analysis of GE-treated worms (10 and 20 µg/mL) compared with untreated control worms. (**B**) Worm median survival of untreated and GE-treated nematodes. Significance refers to the effect of GE with respect to untreated control worms (Mantel–Cox test, ** *p* < 0.01). (**C**) Microscope detection of the lipofuscin aging pigment in control and GE-treated worms. (**D**) Quantification of lipofuscin aging pigment in GE-treated worms compared with untreated control worms (mean ± SD). Significance refers to the effect of GE with respect to untreated control worms (Student’s *t*-test, *** *p* < 0.001).

For this reason, we aimed to determine if the anti-obesity and antioxidant activities of GE could be accompanied by an improvement in the *C. elegans* life expectancy and aging. For this purpose, we initially evaluated the effect of treatment with GE (20 µg/mL) on *C. elegans* lifespan, in comparison with untreated controls. As can be observed in Figure 6A, GE induced a significant increase in the life expectancy of N2 worms, suggesting the life-prolonging activity of the BACs found in GE in this nematode. In this experiment, while the median survival of worms from the NGM and GE 10 µg/mL-treated groups was 17 days, it was 20 days for the worms treated with 20 µg/mL of GE (*p* = 0.0083, Figure 6B).

We then monitored the intestinal accumulation of lipofuscin, a pigment related to aging [71]. Thus, treatment with 20 µg/mL of GE induced a significant reduction in the pigment lipofuscin (Figure 6C,D), suggesting that the antioxidant and life-prolonging activities of GE were accompanied by the anti-aging activity of this extract in *C. elegans*.

Different works have reported the antioxidant, anti-aging, and life-prolonging properties of bioactive compounds contained in different mushrooms using the *C. elegans* model. Thus, a mycelial water extract (MWE) from *Cordyceps sobolifera* exhibited antiaging and antioxidant properties in *C. elegans* [72]. Moreover, treatment with three different extracts from *Lignosus rhinoceros* induced antioxidant properties in *C. elegans*, together with an extended lifespan and a reduction in lipofuscin [64]. This study revealed that these health-promoting effects were mediated through the DAF-16/FOXO signalling pathway, although SKN-1 was not affected by this extract. Taken together, our data demonstrate that the anti-obesity and antioxidant activities of GE are mediated through the activation of the daf-16/FOXO and skn-1/nrf-2 signalling pathways, respectively, which in turn increase *C. elegans* life expectancy and reduce aging.

## 4. Conclusions

In conclusion, our *Grifola frondosa* (GE) extract has been demonstrated to represent an important source of polysaccharides and phenolic compounds with in vitro antioxidant activity. The extract of GE was considered nongenotoxic in a preliminary SOS/umu screening test. The in vivo evaluation demonstrated that treatment with GE significantly reduced the lipid content in *C. elegans*, decreased the intracellular ROS accumulation and the aging-related lipofuscin pigment, and increased the nematode lifespan. Mutant and gene expression analyses revealed that the activation of the *DAF-16/FOXO* and *SKN-1/NRF-2* signalling pathways was necessary for the anti-obesity and antioxidant activities of our GE extract, respectively. Although further research is needed to demonstrate the effect of this GE extract in a mammalian model of obesity, our findings would suggest the potential use of this extract as a functional ingredient in the prevention of metabolic syndrome-related diseases.

## Figures and Tables

**Table 1:** Characterization of the GE extract composition. Mean value ± standard deviation.

	% Total Weight
Yield (wt %) ^a^	6.6 ± 0.5
Ash (wt %)	7.2 ± 0.2
Protein (wt %)	23.1 ± 0.1
Carbohydrates ^b^ (wt %)	38.3 ± 3.4
of which	
Glucose (β-glucan)	20.9 ± 2.6
Galactose	5.6 ± 0.4
Mannose	5.2 ± 0.3
Fucose	5.1 ± 0.1
Glucuronic acid	0.8 ± 0.2
Xylose	0.3 ± 0.1
Other ^c^	<0.5
Total Phenolics (mg GAE/g)	25.9 ± 0.2
TEAC (µg TE/g)	55.3 ± 1.4

^a^ on a dry basis; ^b^ estimated as the sum of all detected monosaccharide units; ^c^ corresponding to rhamnose, arabinose, N-acetylglucosamine, and galacturonic acid, only present in trace amounts or not detected.

## Supplementary Materials

The following are available online at https://www.mdpi.com/article/10.3390/nu13113968/s1, Table S1: Gene expression probes used for the quantitative real-time PCR analysis.
