# Supplementary material for: Grifola frondosa (Maitake) Extract Reduces Fat Accumulation and Improves Health Span in C. elegans through the DAF-16/FOXO and SKN-1/NRF2 Signalling Pathways
Source: Nutrients. 2021 Nov 7;13(11):3968. doi: 10.3390/nu13113968 (PMC8620745; doi:10.3390/nu13113968)
Supplement: Supplementary file 1 [file nutrients-13-03968-s001.zip › nutrients-1393989-supplementary.pdf]

Supplementary material

Table S1. Gene expression probes used for the quantitative real time PCR analysis.

Gene	Description	Fw sequence	Rv sequence	Probe sequence
fat-7 *	Delta(9)-fatty-acid desaturase fat-7	ACAAGTTAAGGAGCATGGAGG	TCTCCTTCCAGAAATAAACGGG	/56-FAM/CAGAGAAAG/Zen/CACTATTTCCCACTGGTCA/3IABkFQ/
fat-5 *	Delta(9)-fatty-acid desaturase fat-5	CGTGTAGAAGGCGATGAAGG	GATTTGTACGAGGATCCGGTG	/56FAM/ACGACTGGA/Zen/ATGAAGGTGGGCA/3IABkFQ/
acox-1 *	Acyl-coenzyme A oxidase	AGCAAACTGGAAAGCGTAGG	AACAAGATACTCGGCAGTGAG	/56FAM/CGGGCGATG/Zen/AATGGGAAGAGACG/3IABkFQ/
maoc-1 *	MAO-C-like dehydratase domain	CAGTAACCAATGTTTGTCCTGG	TCATGGATTGTGCAGTCTGG	/56FAM/CTGCTTGGG/Zen/CTGGAAATGATTCTGAC/3IABkFQ/
**Gene**	**Description**	**TaqMan Reference Gene Expression assay**		
daf-16 **	Fork-head domain-containing protein;Forkhead box protein O	Ce02422838_m1		
daf-2 **	Insulin-like receptor subunit beta; Protein kinase domain-containing protein; Receptor protein-tyrosine kinase	Ce02444349_m1		
sod3 **	Superoxide dismutase 3	Ce02404515_g1		
xbp1 **	BZIP domain-containing protein	Ce02421281_g1		
hsp4 **	Endoplasmic reticulum chaperone BiP homolog; Heat shock 70 kDa protein D	Ce02434874_g1		
ire1 **	Endoribonuclease;Serine/threonine-protein kinase	Ce02435651_g1		
skn-1 **	BZIP domain-containing protein; Protein skinhead-1	Ce02407447_g1		
pmp-3 **	Peroxisomal Membrane Protein related	Ce02485188_m1		

* PrimeTime qPCR Probe Assays (Integrated DNA Technologies, Inc).

** TaqMan® Gene Expression Assays (Thermo Fisher Scientific).
